# Incidence, Risk Factors, and Mortality Associated With Second Malignant Neoplasms Among Survivors of Adolescent and Young Adult Cancer

**DOI:** 10.1001/jamanetworkopen.2019.5536

**Published:** 2019-06-07

**Authors:** Chun Chao, Smita Bhatia, Lanfang Xu, Kimberly L. Cannavale, F. Lennie Wong, Po-Yin Samuel Huang, Robert Cooper, Saro H. Armenian

**Affiliations:** 1 Department of Research and Evaluation, Kaiser Permanente Southern California, Pasadena; 2 Institute for Cancer Outcomes and Survivorship, School of Medicine, University of Alabama at Birmingham; 3 MedHealth Statistical Consulting Inc, Solon, Ohio; 4 Department of Population Sciences, City of Hope, Duarte, California; 5 Department of Family Medicine, Los Angeles Medical Center, Kaiser Permanente Southern California, Los Angeles; 6 Department of Pediatric Oncology, Los Angeles Medical Center, Kaiser Permanente Southern California, Los Angeles

## Abstract

**Importance:**

Detailed data describing the epidemiology of second malignant neoplasms (SMN) are needed for survivors of adolescent and young adult (AYA) cancer to inform the development of age-appropriate survivorship care guidelines.

**Objective:**

To describe the incidence, risk factors, and mortality for SMN in survivors of AYA cancer.

**Design, Setting, and Participants:**

This retrospective matched cohort study included 10 574 two-year survivors diagnosed with cancer between January 1, 1990, and December 31, 2012, at age 15 to 39 years in an integrated health care delivery system in Southern California. A comparison cohort without a history of cancer was individually matched 13:1 to survivors of AYA cancer by age, sex, and calendar year. Data analysis was completed in July 2018.

**Exposures:**

Secondary malignant neoplasm risk factors of interest included age, stage, and calendar year at first cancer diagnosis; sex; race/ethnicity; radiation therapy; and chemotherapy.

**Main Outcomes and Measures:**

Diagnoses of SMN were ascertained using cancer registries from the National Cancer Institute Surveillance, Epidemiology, and End Results Program through December 31, 2014. Poisson regression was used to evaluate the association between cancer survivor status and developing SMN and risk factors for SMN, while risk of all-cause mortality by SMN status was examined in Cox regression.

**Results:**

A total of 10 574 survivors of AYA cancer (6853 [64.8%] female; median [range] age, 33 [15-39] years; 622 with SMN) and 136 683 participants in the comparison cohort (88 513 [64.8%] female; median [range] age, 33 [15-39] years; 3437 with first cancer) were included. In survivors of AYA cancer, 20-year cumulative incidence of SMN was 12.5%. The incidence rate ratio (IRR) of developing SMN in survivors of AYA cancer was 2.6 (95% CI, 2.4-2.9) compared with the comparison cohort. Survivors of breast cancer, melanoma, and testicular cancer had substantially elevated risk for SMN of the same organ (IRR, 5.6 [95% CI, 4.6-6.8], 11.2 [95% CI, 7.3-17.2], and 16.2 [95% CI, 6.8-38.4], respectively). Among survivors of AYA cancer, older age (IRR for age 30-39 years, 1.79 [95% CI, 1.21-2.65]), female sex (IRR, 1.31 [95% CI, 1.09-1.57]), white race/ethnicity (IRR for Asian race, 0.61 [95% CI, 0.43-0.87]), advanced stage at first cancer diagnosis (IRR for stage II, 1.29 [95% CI, 1.11-1.65]), and use of radiotherapy (IRR, 1.50 [95% CI, 1.26-1.79]) were associated with increased risk of SMN. Survivors of AYA cancer who developed SMN had an all-cause mortality rate 7.2 (95% CI, 6.1-8.5) times greater than survivors without SMN.

**Conclusions and Relevance:**

This study suggests that SMN risk is elevated in survivors of AYA cancer and varies across survivor subgroups. Survival following SMN may be significantly compromised. These data may form the basis for identifying individuals at high risk, as well as informing screening for SMN.

## Key Points

**Question:**

What are the incidence patterns and risk factors for second malignant neoplasms (SMN) among survivors of adolescent and young adult cancer?

**Findings:**

This cohort study of 10 574 survivors of adolescent and young adult cancer found that these individuals had a 2.6-fold greater risk of developing SMN compared with individuals with no history of cancer. Risk factors for SMN included demographic factors, stage at diagnosis, and radiation therapy and varied by first cancer type, with survivors of breast cancer, melanoma, and testicular cancer having substantially elevated risk for SMN of the same type.

**Meaning:**

This study suggests that SMN risk is elevated in survivors of adolescent and young adult cancer and provides data that may be used to identify individuals at high risk and inform screening for SMN.

## Teaser

This cohort study estimates the incidence, mortality, and associated risk factors for the development of second malignant neoplasms in individuals who survived adolescent and young adult cancers.

## Introduction

Second malignant neoplasms (SMN) are among the most debilitating late effects in cancer survivors.^1,2^ In survivors of childhood cancers, SMN is the most common cause of non–relapse-related mortality.^3^ In an analysis using the National Cancer Institute’s Surveillance, Epidemiology, and End Results (SEER) data, the majority of individuals with 2 primary cancers died of their SMN rather than their initial cancer.^4^ Therefore, prevention and early detection of SMN are critical for prolonging life for cancer survivors.^5,6^

Extensive effort has been made to characterize SMN in survivors of childhood cancer.^7,8,9,10,11^ However, this knowledge may not be applicable to survivors of cancers diagnosed later in life. The risk and risk factors for SMN vary across age groups because of differences in primary cancers, treatment exposures, age at exposures, exposure to other etiological factors, and background incidence,^12^ necessitating age-specific approaches for managing SMN risk among cancer survivors.

Adolescents and young adults (AYAs; defined as individuals aged 15-39 years) have been an understudied population for cancer survivorship. Several studies have evaluated SMN risk by age groups^13,14,15^ or in survivors of cancer types that are more common in AYAs.^16,17,18,19,20^ Most of these studies have examined data from large cancer registries such as SEER and used an external comparison group (eg, the general population) for evaluating the excess risk of SMN. However, using external comparison for causal inference is associated with several known limitations,^21^ including the inability to address potential incomparability between cancer survivors and the general population, such as insurance status and health care access.^22^ Information on therapeutic agents is also not typically available in these cancer registries. Furthermore, detailed characterization of timing and risk factors for SMN in survivors of AYA cancer remain largely incomplete in the literature.

In the current study, we evaluated the development of SMN in survivors of AYA cancer who were members of Kaiser Permanente Southern California (KPSC) using a matched cohort design, providing an internal comparison with similar demographic characteristics, socioeconomic status, and access to health care. We used existing electronic medical records to examine the association between treatment exposures and SMN in survivors of AYA cancer. Our objective was to provide a comprehensive assessment of SMN risk in survivors of AYA cancer using study methods that minimized confounding to inform survivorship care planning for survivors of AYA cancer.

## Methods

### Study Setting and Study Population

Kaiser Permanente Southern California is an integrated health care organization that provides comprehensive health services to more than 4.4 million racially/ethnically and socioeconomically diverse members (approximately 1% of the US population) who are broadly representative of residents in Southern California.^23,24^ Members of KPSC who met the following criteria were included in the cohort of survivors of AYA cancer: (1) diagnosed with invasive cancer at age 15 to 39 years between 1990 and 2012 at KPSC; (2) survived for at least 2 years (index date) after cancer diagnosis; and (3) retained KPSC membership at index date. Among individuals who met the inclusion criteria, those diagnosed with another primary cancer before the index date were excluded, as we were mainly interested in SMN that occurred after completion of treatment to inform posttreatment surveillance strategies. We identified survivors of AYA cancer using KPSC’s SEER-affiliated cancer registry. Quality of the cancer registry data is assured by the SEER standard and is audited by SEER staff on a regular basis.^25^

Members of KPSC without a history of cancer were included as a reference group (referred to as the *comparison cohort* in this article) to survivors of AYA cancer. Participants in the comparison cohort were matched 13:1 to each individual cancer survivor by age (yearly), sex, and calendar year of the index date. They were identified from those who were KPSC members in the year of the corresponding cancer diagnosis for a cancer survivor and who survived and remained as a KSPC member 2 years after (ie, after the index date). This study was approved, and the requirement of informed consent waived, by KPSC’s institutional review board. This article follows the Strengthening the Reporting of Observational Studies in Epidemiology (STROBE) reporting guideline.^26^

### Identification of SMN

All study participants were followed up from index date to death or end of 2014 (for SMN or first cancer) or end of 2015 (for mortality outcome), whichever came first. Diagnoses of malignant neoplasms, including those made outside of the KPSC system (eg, if a participant terminated KPSC membership during study follow-up), were identified using both KPSC’s cancer registry and the California State Cancer Registry. Both registries are SEER-affiliated and do not capture relapse or metastasis. As a result, a second cancer record for the same participant was considered SMN. For SMN of the same organ or type as the first cancer, SEER used a set of multiple primaries rules to distinguish SMN from recurrence.^27,28^ These rules differ by cancer types. For solid tumors, the determining factors may involve histology, biology, or clinical presentation, among other factors, while for hematologic cancers, the rules were mainly based on histology. The SEER multiple primaries rules for the most common first cancer types in survivors of AYA cancer are illustrated in eTable 1 in the Supplement. Nonmelanoma skin cancer was not included in this study as it was not consistently captured by the cancer registries.

### Data Collection

Covariates of interest included demographic characteristics, cancer characteristics, exposure to chemotherapy and radiation therapy, and death information. All data for this study were collected using KPSC’s electronic health records and cancer registries, except for information on death, which was also collected using outside claims, California State Death Files, and national Social Security death files. Specifically, race/ethnicity information was obtained from KPSC’s membership file and the cancer registry.

### Statistical Analysis

The distribution of demographic, cancer, and treatment characteristics and the incidence rate of subsequent cancer were calculated. The crude and the adjusted incidence rate ratios (IRRs) of SMN associated with being a survivor of AYA cancer compared with those in the comparison cohort were estimated using bivariate and multivariable Poisson regression adjusting for age, sex (except for breast, cervix, ovary, and testicular cancer), and race/ethnicity, for the overall study cohort and by patient characteristics, first cancer type, and SMN type. We also calculated IRRs for specific SMN among the most common first cancer types in survivors of AYA cancer (ie, breast, lymphoma, melanoma, and testicular cancer). For breast SMN among breast cancer survivors, a sensitivity analysis was performed including only breast second primary malignant neoplasms of different histology or laterality (about 70% of all identified breast SMN) from the first cancer. All testicular second primary malignant neoplasms identified in this study had different laterality from the first testicular cancer.

The cumulative incidence of SMN over the study follow-up period was calculated using nonparametric methods accounting for competing risk and presented graphically for visual inspection, for the study cohort and by selected common first cancer type.^29^ Differences in cumulative incidence were tested using the Gray test.^30^

Among survivors of AYA cancer, multivariable Poisson regression was performed to evaluate the association of age, sex, race/ethnicity, first cancer type, TNM stage at diagnosis, and exposure to radiation therapy on risk of SMN, overall and separately with solid vs nonsolid SMN (defined as lymphomas, leukemias, and myelomas). The association of initial cancer type was evaluated in separate analyses restricted to the more prevalent cancer types, ie, breast cancer, lymphoma, testicular cancer, melanoma, and thyroid cancer. The associations of exposures to selected chemotherapy agents (alkylating agents, anthracycline, platinum, and epipodophyllotoxins) were evaluated in another analysis restricted to those diagnosed between 2000 and 2012, when chemotherapy data became accessible electronically.

Risk factors for SMN were also evaluated separately for the most common first cancer types among survivors of AYA cancer. As sample size allowed, risk factor analyses were also conducted for breast SMN among breast cancer survivors and melanoma SMN among melanoma survivors.

For the overall risk factor analyses, sensitivity analyses excluding breast SMN and, separately, melanoma SMN as outcomes were conducted to further understand whether these SMN might be driving the associations observed given that they were the 2 most common SMN types.

The increase in all-cause mortality after a diagnosis of SMN in survivors of AYA cancer relative to that after the first cancer diagnosis in the comparison cohort was examined in bivariate and multivariable Cox models adjusting for age, sex, and race/ethnicity. Among survivors of AYA cancer, the risk of all-cause mortality associated with having an SMN was evaluated using a time-dependent Cox model to account for the time-varying nature of SMN diagnosis, adjusting for age, sex, race/ethnicity, stage at diagnosis, and cancer type. All analyses in this study were completed by July 2018 with SAS statistical software version 9.3 (SAS Institute Inc). A 2-sided *P* value less than .05 was considered statistically significant.

## Results

A total of 14 753 KPSC members between ages 15 and 39 years were diagnosed with invasive cancer during 1990 to 2012 at KPSC. Of these, 12 994 (88%) survived at least 2 years and were eligible for this study. After applying the exclusion criteria, 10 574 survivors of AYA cancer (6853 [64.8%] female; median [range] age, 33 [15-39] years; 622 with SMN) were included in the analyses with 136 683 participants (88 513 [64.8%] female; median [range] age, 33 [15-39] years; 3437 with first cancer) in the matched comparison cohort (eFigure 1 in the Supplement). Among survivors of AYA cancer, 7% were aged 15 to 19 years; 24%, 20 to 29 years; and 68%, 30 to 39 years. Approximately 47% were non-Hispanic white. The most common first cancer diagnosis was breast cancer (17%), followed by thyroid cancer (14%), melanoma (11%), lymphomas (11%), and testicular cancer (9%). One-quarter (25%) of survivors of AYA cancer received external beam radiation therapy. In survivors of AYA cancer diagnosed in 2000 to 2012, exposure to selected chemotherapy agents ranged from 7% for epipodophyllotoxins to 24% for anthracyclines (Table 1).

**Table 1.** Distribution of Demographic and Cancer Characteristics of Study Participants

Characteristic	No. (%)		*P* Value
	Survivors of AYA Cancer (n = 10 574)	Comparison Cohort (n = 136 683)	
Age, y			
Median (range)	33 (15-39)	33 (15-39)	.96
15-19	768 (7.3)	9936 (7.3)	>.99
20-29	2580 (24.4)	33 337 (24.4)	
30-39	7226 (68.3)	93 410 (68.3)	
Female	6853 (64.8)	88 513 (64.8)	.91
Race/ethnicity			
Non-Hispanic white	4921 (46.5)	40 035 (29.3)	<.001
Asian or Pacific Islander	972 (9.2)	11 665 (8.5)	
Non-Hispanic black	922 (8.7)	13 550 (9.9)	
Hispanic	3717 (35.2)	47 690 (34.9)	
Other or unknown	42 (0.4)	23 743 (17.4)	
Length of membership at index, mean (SD), y	7.7 (6.4)	7.4 (6.1)	<.001
Length of follow-up, y			
Mean (SD)	8.8 (6.5)	1.1 (6.5)	<.001
Median (range)	7.7 (0-23.0)	9.6 (0-23.0)	
Years of diagnosis			
1990-1999	3796 (35.9)		
2000-2012	6778 (64.1)		
Cancer type			
Anus or rectum	187 (1.8)		
Bladder	30 (0.3)		
Bones and joints	111 (1.1)		
Brain and other nervous system	399 (3.8)		
Breast^a^	1810 (17.1)		
Cervix	489 (4.6)		
Colon	225 (2.1)		
Ill-defined or unspecified	141 (1.3)		
Kaposi sarcoma	127 (1.2)		
Lymphocytic leukemia	120 (1.1)		
Lymphoma	1124 (10.6)		
Non-Hodgkin	510 (4.8)		
Hodgkin	614 (5.8)		
Melanoma	1187 (11.2)		
Multiple myeloma	41 (0.4)		
Myeloid leukemia	204 (1.9)		
Oral cavity and pharynx	231 (2.2)		
Other			
Digestive system	153 (1.5)		
Endocrine system	37 (0.4)		
Female genital system	37 (0.4)		
Leukemia	7 (0.1)		
Male genital system	24 (0.2)		
Skin	166 (1.6)		
Ovary	418 (4.0)		
Respiratory system	117 (1.1)		
Soft tissue, including heart	238 (2.3)		
Testis	914 (8.6)		
Thyroid	1501 (14.2)		
Urinary system	235 (2.2)		
Uterus	301 (2.8)		
TNM stage			
NA^b^	1388 (13.1)		
I	5165 (48.9)		
II	2180 (20.6)		
III	999 (9.5)		
IV	446 (4.2)		
Unknown	396 (3.8)		
Radiation therapy			
Yes	3620 (34.2)		
Excluding thyroid cancer^c^	2607 (24.7)		
Chemotherapy in 2000-2012 (n = 6778 surviving patients)			
Any of the selected agents below			
Alkylating agents	1414 (20.9)		
Anthracyclines	1623 (24.0)		
Epipodophyllotoxin	504 (7.4)		
Platinums	899 (13.3)		

Abbreviations: AYA, adolescent and young adult; NA, not applicable.

^a^ Includes male (n = 4) and female (n = 1806) breast cancer.

^b^ TNM stage was NA for some cancer types, such as leukemia or brain cancer.

^c^ Thyroid cancer was excluded because most patients with thyroid cancer who received radiotherapy received radioactive iodine instead of external beam radiation.

### Risk of SMN

The survivors of AYA cancer and the comparison cohort contributed 93 290 and 1 379 136 person-years of observation, respectively, through December 31, 2014. During the study period, 622 survivors of AYA cancer developed an SMN (6.7 per 1000 person-years). The 10- and 20-year cumulative incidence of SMN from index date was 5.6% and 12.5%, respectively. Of survivors of AYA cancer who developed SMN, the most common SMN types were breast cancer (32%), melanoma (14%), and ovarian cancer (5%). Ninety-three percent of the SMN were solid tumors (Table 2). As a group, gastrointestinal cancers also constituted a substantial proportion (11%) of SMN (eTable 2 in the Supplement). The median (range) follow-up time in survivors of AYA cancer was 7.7 (0-23.0) years from the index date, or 9.7 (2-25) years after initial cancer diagnosis.

**Table 2.** Incidence and Adjusted IRR Comparing SMN Among Survivors of AYA Cancer and Matched Comparison Cohort

Demographic and Cancer Characteristic	Survivors of AYA Cancer				Comparison Cohort		Multivariable Poisson Model^a^	
	Second Primary Cancer, No.	Cancer Survivors, No.	Person-Years	Incidence/1000 Person-Years	First Primary Cancer, No.	Incidence/1000 Person-Years	IRR (95% CI)	*P* Value
Overall	622	10 574	93 290	6.67	3437	2.49	2.63 (2.42-2.87)	<.001
Age at first cancer diagnosis in cancer survivors, y								
15-19	29	768	6801	4.26	56	0.57	7.50 (4.79-11.76)	<.001
20-29	102	2580	22 793	4.48	415	1.26	3.45 (2.78-4.29)	<.001
30-39	491	7226	63 697	7.71	2966	3.12	2.42 (2.20-2.66)	<.001
Sex								
Male	184	3721	33 146	5.55	734	1.50	3.57 (3.04-4.21)	<.001
Female	438	6853	60 144	7.28	2703	3.04	2.37 (2.14-2.62)	<.001
Race/ethnicity								
Non-Hispanic white	337	4921	47 538	7.09	1388	3.28	2.26 (2.01-2.55)	<.001
Non-Hispanic black	65	922	8210	7.92	442	3.02	2.67 (2.06-3.46)	<.001
Hispanic	181	3717	29 220	6.19	896	2.18	2.96 (2.52-3.47)	<.001
Asian or Pacific Islander	37	972	7900	4.68	325	3.26	1.41 (1.00-1.98)	.05
By first cancer type in survivors of AYA cancer								
Breast (female)	181	1806	14 889	12.16	926	3.66	3.30 (2.81-3.87)	<.001
Hodgkin lymphoma	33	614	5832	5.66	139	1.73	3.10 (2.12-4.55)	<.001
Non-Hodgkin lymphoma	26	510	4348	5.98	119	1.85	3.17 (2.07-4.86)	<.001
Melanoma	101	1187	12 468	8.10	441	2.55	3.00 (2.38-3.79)	<.001
Testicular	31	914	9035	3.43	166	1.39	2.38 (1.61-3.51)	<.001
Thyroid	39	1501	13 146	2.97	409	2.39	1.23 (0.88-1.71)	.22
Brain	12	346	2391	5.02	77	1.77	2.76 (1.49-5.08)	.001
Cervix	21	489	4957	4.24	200	2.84	1.48 (0.94-2.32)	.09
Ovary	19	418	3858	4.92	146	2.63	1.80 (1.12-2.91)	.02
By SMN type^b^								
Solid SMN	578	10 574	93 290	6.20	3216	2.33	2.61 (2.39-2.85)	<.001
Nonsolid SMN	44	10 574	93 290	0.47	221	0.16	3.02 (2.18-4.18)	<.001
Breast (female)	198	6853	60 144	3.29	1115	1.25	2.61 (2.24-3.04)	<.001
Melanoma	85	10 574	93 290	0.91	301	0.22	3.20 (2.51-4.07)	<.001
Lymphoma^c^	28	10 574	93 290	0.30	139	0.10	3.05 (2.03-4.58)	<.001
Leukemia	14	10 574	93 290	0.15	60	0.04	3.55 (1.98-6.37)	<.001
Central nervous system	15	10 574	93 290	0.16	136	0.10	1.64 (0.96-2.79)	.07
Gastrointestinal	70	10 574	93 290	0.75	386	0.28	2.75 (2.13-3.54)	<.001
Bladder	5	10 574	93 290	0.05	28	0.02	2.60 (1.00-6.77)	.05
Bone	7	10 574	93 290	0.08	9	0.01	11.41 (4.25-30.65)	<.001
Cervix	6	6853	60 144	0.10	93	0.10	0.95 (0.42-2.18)	.91
Ovary	28	6853	60 144	0.47	96	0.11	4.24 (2.78-6.47)	<.001
Uterus	15	6853	60 144	0.25	164	0.18	1.35 (0.79-2.29)	.27
Other female genital	10	6853	60 144	0.17	39	0.04	3.81 (1.90-7.64)	<.001
Lung	12	10 574	93 290	0.13	85	0.06	2.02 (1.10-3.71)	.02
Oropharynx	18	10 574	93 290	0.19	68	0.05	3.54 (2.10-5.97)	<.001
Testis	17	3721	33 146	0.51	36	0.07	6.41 (3.57-11.49)	<.001
Prostate	16	3721	33 146	0.48	119	0.24	2.10 (1.24-3.55)	.01
Renal	19	10 574	93 290	0.20	101	0.07	2.84 (1.74-4.65)	<.001
Soft tissue	10	10 574	93 290	0.11	27	0.02	5.44 (2.62-11.31)	<.001
Thyroid	23	10 574	93 290	0.25	218	0.16	1.52 (0.99-2.34)	.06
Other	26	10 574	93 290	0.28	215	0.16	1.88 (1.25-2.82)	.002

Abbreviations: AYA, adolescent and young adult; IRR, incidence rate ratio; SMN, second malignant neoplasm.

^a^ Adjusted for age, sex (if applicable), and race/ethnicity.

^b^ The SMN type applies to SMN for survivors of AYA cancer and to the first cancer developed in the comparison cohort.

^c^ Includes patients with Hodgkin lymphoma and 24 patients with non-Hodgkin lymphoma.

### Excess Risk of SMN in Survivors of AYA Cancer

Survivors of AYA cancer had a 2.6-fold (95% CI, 2.4-2.9) increased risk of subsequent malignant neoplasm compared with the comparison cohort (Table 2). The IRR was significantly elevated for all demographic subgroups and was greatest for those aged between 15 and 19 years at first cancer diagnosis (adjusted IRR [aIRR], 7.5 [95% CI, 4.8-11.8]). The aIRR for SMN was significantly elevated for all primary cancer types, except for thyroid and cervical cancer. The aIRR for SMN was also significantly elevated for most SMN cancer types, except for cancer of the cervix, uterus, central nervous system, and thyroid. Risk for SMN was highest for bone SMN (aIRR, 11.4 [95% CI, 4.3-30.7]).

The IRR for specific pairs of first and second cancer showed unique SMN patterns across different first cancer types (Table 3). Survivors of breast cancer, melanoma, and testicular cancer had 5.62 (95% CI, 4.63-6.83), 11.22 (95% CI, 7.34-17.16), and 16.17 (95% CI, 6.80-38.43) times greater risk of developing another breast cancer, melanoma, and testicular cancer, respectively, compared with the comparison cohort. In the sensitivity analysis, aIRR for developing another breast cancer of different histology or laterality among breast cancer survivors was 4.3 (95% CI, 3.5-5.4). Breast cancer survivors also had elevated risk for subsequent ovarian cancer (aIRR, 5.3 [95% CI, 2.7-10.4]) and melanoma (aIRR, 2.95 [95% CI, 1.45-6.00]). Risk for developing a breast SMN was elevated in lymphoma and melanoma survivors (aIRR, 2.31 [95% CI, 1.18-4.51] and aIRR, 1.80 [95% CI, 1.04-3.12], respectively). Survivors of testicular cancer were also at increased risk of subsequent prostate cancer (aIRR, 2.9 [95% CI, 1.2-7.2]).

**Table 3.** Incidence and Adjusted IRR Comparing Subsequent Cancer Among Survivors of AYA Cancer and Matched Comparison Group

First Cancer Type in Survivors of AYA Cancer	Survivors of AYA Cancer			Comparison Cohort		Multivariable Poisson Model	
	SMN, No.	Incidence/1000 Person-Years	Time From First Cancer to SMN Diagnosis	First Cancer, No.^a^	Incidence/1000 Person-Years	IRR (95% CI)	*P* Value
**Breast Cancer**							
Subsequent cancer type during study follow-up							
Solid tumor	174	11.69	8.82 (2.01-23.83)	885	3.50	3.32 (2.82-3.91)	<.001
Nonsolid tumor	7	0.47	12.38 (2.24-23.07)	41	0.16	2.88 (1.29-6.42)	.01
Breast	135	9.07	8.88 (2.01-23.83)	406	1.60	5.62 (4.63-6.83)	<.001
Breast of different histology or laterality	105	6.99	7.94 (2.01-23.84)	406	1.60	4.34 (3.50-5.38)	<.001
Melanoma	9	0.60	7.78 (2.49-15.41)	49	0.19	2.95 (1.45-6.00)	.003
Ovary	11	0.74	10.33 (4.31-21.52)	35	0.14	5.28 (2.68-10.39)	<.001
**Lymphoma**							
Subsequent cancer type during study follow-up							
Solid tumor	46	4.52	11.80 (2.29-23.06)	240	1.66	2.61 (1.90-3.58)	<.001
Nonsolid tumor	13	1.28	8.49 (2.39-16.66)	18	0.12	11.20 (5.47-22.91)	<.001
Breast	10	2.04	15.45 (2.29-22.96)	62	0.91	2.31 (1.18-4.51)	.01
Non-Hodgkin lymphoma	10	0.98	8.07 (2.59-16.66)	7	0.05	22.57 (8.57-59.43)	<.001
**Melanoma**							
Subsequent cancer type during study follow-up							
Solid tumor	96	7.70	7.65 (2.07-24.06)	413	2.39	3.03 (2.39-3.85)	<.001
Nonsolid tumor	5	0.40	7.12 (3.59-16.92)	28	0.16	2.53 (0.91-6.99)	.07
Breast	16	2.09	15.36 (2.36-24.06)	124	1.19	1.80 (1.04-3.12)	.03
Melanoma	59	4.73	6.13 (2.07-21.72)	41	0.24	11.22 (7.34-17.16)	<.001
**Testicular Cancer**							
Subsequent cancer type during study follow-up							
Solid tumor	31	3.43	8.80 (2.19-22.64)	148	1.24	2.66 (1.80-3.94)	<.001
Nonsolid tumor	0	0	NA	18	0.15	NA	NA
Prostate	6	0.66	18.98 (12.13-21.35)	26	0.22	2.90 (1.16-7.21)	.02
Testis^b^	13	1.44	5.75 (2.19-14.15)	9	0.08	16.17 (6.80-38.43)	<.001

Abbreviations: AYA, adolescent and young adult; IRR, incidence rate ratio; NA, not applicable; SMN, second malignant neoplasm.

^a^ This column shows the number of first cancers in the comparison cohort that were of the same cancer type as those presented as SMN in survivors of AYA cancer.

^b^ All testicular SMN as identified by the Surveillance, Epidemiology, and End Results multiple primary rules in this study were of different laterality.

### Cumulative Incidence Function of SMN

The trajectory of overall SMN cumulative incidence functions varied by first cancer type (eFigure 2 in the Supplement). In breast cancer survivors, cumulative incidence increased steadily over the follow-up period. By contrast, a steeper increase in incidence was observed 18 to 20 years after diagnosis in lymphoma, melanoma, and testis cancer survivors.

The Figure shows the cumulative incidence functions by specific pairs of first (breast, melanoma, testis) and second cancer. Increased risk of SMN of the same type as the first cancer, but not for other SMN types examined, was apparent immediately after index date (eg, melanoma SMN among melanoma survivors, shown in Figure, C).

**Figure.**
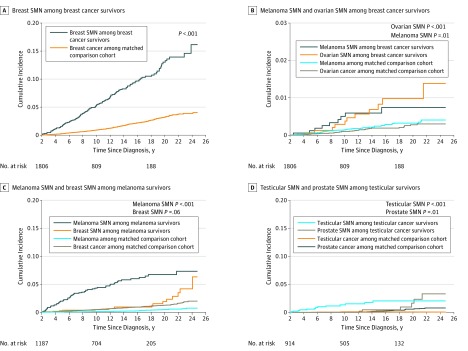
Cumulative Incidence Function Considering Competing Risk Among Survivors of Adolescent and Young Adult Cancer by Second Malignant Neoplasm (SMN) and First Cancer Type The* P* value compares SMN incidence by cancer survivor status using the Fine and Gray method. A different scale for the y axis is used in panel B to allow the visual distinction of curves.

### Risk Factors for SMN

Among survivors of AYA cancer, older age at diagnosis (IRR for age 30-39 years, 2.09 [95% CI, 1.35-3.25]), female sex (IRR, 1.38 [95% CI, 1.14-1.67]), advanced stage at diagnosis (IRR for stage II, 1.31 [95% CI, 1.07-1.60]), and exposure to radiation therapy (IRR, 1.56 [95% CI, 1.30-1.87]) were independently associated with greater risk of solid SMN (Table 4). On the other hand, Asian race/ethnicity (IRR compared with non-Hispanic white, 0.60 [95% CI, 0.41-0.86]), and more recent calendar period of diagnosis (IRR for diagnosis in 2003-2014 compared with 1990-2002, 0.81 [95% CI, 0.67-0.97]) were associated with lower risk of solid SMN. For nonsolid SMN, Hispanic race/ethnicity (IRR, 1.99 [95% CI, 1.01-3.95]) advanced stages at diagnosis (IRR for stage II, 2.58 [95% CI, 1.04-6.43]; IRR for stage III/IV, 4.41 [95% CI, 1.77-11.00]), and cancers for which TMN stage were not applicable (IRR, 5.17 [95% CI, 2.13-12.56]) were associated with higher risk. In sensitivity analysis excluding breast SMN as an outcome, female sex was no longer associated with increased SMN risk (IRR, 0.77 [95% CI, 0.63-0.94]). In sensitivity analyses excluding melanoma SMN, Asian race/ethnicity was no longer associated with reduced risk of solid SMN (IRR, 0.79 [95% CI, 0.55-1.13]).

**Table 4.** Multivariable Poisson Model of Potential Risk Factors for Second Primary Cancer Among Survivors of AYA Cancer

Characteristic	Any SMN		Solid SMN		Nonsolid SMN	
	IRR (95% CI)	*P* Value	IRR (95% CI)	*P* Value	IRR (95% CI)	*P* Value
**All Cancer Survivors Diagnosed 1990-2012**						
Age at diagnosis, y						
15-19	1 [Reference]		1 [Reference]		1 [Reference]	
20-29	1.08 (0.70-1.66)	.73	1.27 (0.79-2.05)	.32	0.38 (0.12-1.19)	.10
30-39	1.79 (1.21-2.65)	<.001	2.09 (1.35-3.25)	.001	0.70 (0.29-1.71)	.44
Sex						
Male	1 [Reference]		1 [Reference]		1 [Reference]	
Female	1.31 (1.09-1.57)	<.001	1.38 (1.14-1.67)	.001	0.70 (0.37-1.33)	.28
Race/ethnicity						
Non-Hispanic white	1 [Reference]		1 [Reference]		1 [Reference]	
Non-Hispanic black	1.00 (0.76-1.32)	>.99	0.96 (0.72-1.28)	.79	1.73 (0.62-4.82)	.29
Hispanic	0.90 (0.75-1.08)	.26	0.84 (0.69-1.02)	.08	1.99 (1.01-3.95)	.05
Asian or Pacific Islander	0.61 (0.43-0.87)	.01	0.60 (0.41-0.86)	.01	0.89 (0.20-3.91)	.87
TNM stage at diagnosis						
I	1 [Reference]		1 [Reference]		1 [Reference]	
NA	1.40 (1.08-1.82)	.01	1.24 (0.94-1.65)	.13	5.17 (2.13-12.56)	<.001
II	1.35 (1.11-1.65)	.003	1.31 (1.07-1.60)	.01	2.58 (1.04-6.43)	.04
III/IV	1.29 (1.00-1.67)	.05	1.17 (0.90-1.54)	.24	4.41 (1.77-11.00)	.002
Calendar period of diagnosis						
1990-2002	1 [Reference]		1 [Reference]		1 [Reference]	
2003-2014	0.80 (0.67-0.96)	.02	0.81 (0.67-0.97)	.02	0.78 (0.40-1.51)	.45
Radiation (yes vs no)	1.50 (1.26-1.79)	<.001	1.56 (1.30-1.87)	<.001	0.95 (0.46-1.96)	.89
**Selected 5 Cancer Type Diagnosed 1990-2012**^a^						
Type of first cancer						
Thyroid	1 [Reference]		1 [Reference]		1 [Reference]	
Breast	2.81 (1.88-4.21)	<.001	2.80 (1.84-4.26)	<.001	2.01 (0.43-9.40)	.37
Lymphoma (Hodgkin and non-Hodgkin)	1.65 (1.04-2.62)	.03	1.38 (0.84-2.27)	.20	4.43 (1.06-18.51)	.04
Melanoma	2.65 (1.79-3.93)	<.001	2.67 (1.78-4.02)	<.001	2.54 (0.55-11.80)	.23
Testis	1.11 (0.64-1.94)	.71	1.31 (0.74-2.34)	.36	Not calculated^b^	NA
**All Cancer Survivors Diagnosed 2000-2012**^c^						
Treatment exposure						
Radiation (yes vs no)	1.14 (0.81-1.59)	.46	1.20 (0.84-1.70)	.31	0.56 (0.14-2.18)	.40
Alkylating agents (yes vs no)	1.24 (0.75-2.07)	.41	1.43 (0.84-2.46)	.19	0.40 (0.08-1.97)	.26
Anthracyclines (yes vs no)	0.96 (0.57-1.63)	.89	0.83 (0.47-1.45)	.51	2.83 (0.61-13.02)	.18
Epipodophyllotoxin (yes vs no)	0.63 (0.27-1.49)	.29	0.61 (0.25-1.52)	.29	0.94 (0.06-15.28)	.96
Platinums (yes vs no)	0.98 (0.53-1.83)	.96	1.01 (0.54-1.91)	.97	0.60 (0.04-10.00)	.72

Abbreviations: AYA, adolescent and young adult; IRR, incidence rate ratio; NA, not applicable; SMN, second malignant neoplasm.

^a^ This regression analysis included survivors of the 5 cancer types only (thyroid cancer, breast cancer, lymphoma, melanoma, and testicular cancer).

^b^ The IRR could not be estimated because of the small sample size in this subgroup.

^c^ Adjusted for age, sex, race/ethnicity, stage at diagnosis, calendar period of diagnosis, and radiation therapy.

In analyses restricted to the 5 most common first cancer types, survivors of breast cancer (aIRR, 2.80 [95% CI, 1.84-4.26]) and melanoma (aIRR, 2.67 [95% CI, 1.78-4.02]) had the greatest increased risk of solid SMN compared with thyroid cancer. Among those whose first cancer was diagnosed in 2000 to 2012, none of the chemotherapy agents examined were significantly associated with SMN, solid or nonsolid (Table 4).

Among breast cancer survivors, Asian individuals had lower risk of developing any SMN (aIRR, 0.50 [95% CI, 0.27-0.93]) or breast SMN, while radiotherapy was associated with increased risk of any SMN (aIRR, 1.52 [95% CI, 1.12-2.05]) or breast SMN (eTable 3 in the Supplement). Among melanoma survivors, advanced stage at diagnosis was associated an almost 3-fold risk of any SMN (aIRR, 2.83 [95% CI, 1.40-5.71]) but not melanoma SMN. Among lymphoma survivors, black race and radiation therapy were associated with increased risk of SMN. Demographic characteristics, stage, and radiation therapy (for testicular cancer) were not associated with risk of SMN among testicular and thyroid cancer survivors (eTable 3 in the Supplement).

### Mortality Following SMN

The 5-year overall mortality following an SMN diagnosis was 31.9% (128 of 401) for survivors of AYA cancer. Adjusted hazard ratio (aHR) for mortality after developing SMN was 1.90 (95% CI, 1.61-2.24) for survivors of AYA cancer compared with mortality after developing first cancer in the comparison group (eTable 4 in the Supplement). Among survivors of AYA cancer, those with SMN were at 7-fold increased risk of dying compared with survivors who did not develop SMN (aHR, 7.17 [95% CI, 6.06-8.49]).

## Discussion

We observed a 3-fold SMN risk increase in survivors of AYA cancer compared with a demographically matched comparison cohort who did not have a history of cancer at the index date. This risk increase was primarily driven by solid SMN. Increased risk for SMN varied by patient characteristics, first cancer type, and SMN type. Among survivors with primary breast cancer, melanoma, and testicular cancer, a particularly elevated risk for SMN of the same organ was found. Overall, we observed several demographic, clinical, and treatment characteristics to be associated with SMN. Furthermore, risk factors for SMN appeared to differ by first cancer type. These findings have important implications for prevention and early detection strategies for SMN and could inform the development of cancer screening guidelines for survivors of AYA cancer.

In general, an SMN risk increase of 4 to 6 times has been reported for childhood cancer survivors compared with the general population.^8,14,31^ However, survivors of AYA cancer bear a greater absolute burden of SMN.^14^ A few studies have specifically examined relative risk of SMN among survivors of AYA cancer, and all used an external group for comparison.^13,14,15,31^ Although previous studies have used different methods, a moderately elevated risk of SMN has been consistently reported.

For survivors of AYA breast cancer, melanoma, and testicular cancer, our data suggest a need for an early detection program for subsequent cancer of the same organ. We used the SEER multiple primary rules established by expert consensus to determine SMN of the same organ in this observational study. While potential misclassification of second primary vs recurrence is possible, a more conservative IRR estimate of developing breast SMN of different histology or laterality among breast cancer survivors resulted in a similar conclusion, suggesting that the implication of our findings for early surveillance is likely valid. The association observed between breast and ovarian cancer as well as between breast cancer and melanoma is consistent with patterns of a shared genetic predisposition (eg, *BRCA1* and/or *BRCA2*).^32,33^ Increased risk of prostate cancer among testicular cancer survivors has not been commonly reported and should be further studied accounting for prostate cancer screening practices.

In our study, female survivors were at greater risk of SMN compared with male survivors, likely because breast cancer was the most common SMN type in this age group. In fact, in a sensitivity analysis excluding breast SMN, female participants were at decreased risk of SMN. Similarly, lower risk in Asian participants for solid SMN could be partially due to the lower risk of melanoma in Asian populations,^34^ as, in the sensitivity analysis excluding melanoma SMN, Asian individuals were no longer at lower risk of SMN. Recent calendar period was associated with lower risk of solid SMN, which could be due to the longer latency of solid SMN. The potential reason for higher risk of nonsolid SMN in Hispanic participants is unclear. We did not observe an association with SMN risk, solid or nonsolid, for any of the chemotherapy categories examined. Although the follow-up time for the chemotherapy analysis was considered limited for evaluating risk of solid tumor SMN, it was reasonable for evaluating nonsolid SMN as most nonsolid malignancies linked to chemotherapy are expected to develop early.

We found different risk factors for SMN by first cancer type, suggesting potentially varying pathogenic mechanisms of SMN by first cancer type. Among breast cancer survivors, despite generally increased breast cancer risk associated with advanced age, younger age at initial diagnosis was not associated with lower risk for subsequent SMN or breast SMN, arguing for a strong genetic predisposition for those diagnosed at young age. As expected, radiotherapy is a risk factor that should be considered for SMN surveillance. In melanoma survivors, advanced stage at diagnosis was associated with an almost 3-fold risk of developing nonmelanoma SMN. Black race and radiation were associated with SMN in lymphoma survivors. The underlying reasons for these observations (except for radiation) are not fully understood.

Those with SMN were at 7-fold increased risk of dying compared with survivors who did not develop SMN. A recent study by Keegan and colleagues^35^ reported worse survival outcomes following SMN compared with those who develop first malignancy of the same type, and this difference was more profound in AYAs than in older adults. A study in breast cancer survivors showed that early detection of the second primary breast cancer was linked to improved relative survival by about 30% to 50%,^5^ supporting implementation of effective screening strategies. The role of traditional cancer risk factors and genetic testing for SMN prevention need to be addressed by future studies.^36,37,38^

### Limitations

There are several limitations that should be considered. In addition to those we have described, cancers diagnosed among patients moving out of California would be missed. We were also unable to perform stratified analyses by more first cancer types or by SMN type owing to limited power. Furthermore, we did not examine the association with radiation field or dose-response relationships. The generalizability of our findings to those without insurance should also be confirmed. Despite the limitations, our study has several unique strengths. First, we used an internal comparison group with equal health care access, eliminating the potential concern of confounding due to differential access and insurance coverage in prior registry-based studies in the United States. Second, we characterized the pairwise relationship between specific first and second cancer type among the most common first cancer types in AYAs, which has implications for SMN prevention and screening. For example, counseling can be offered for female melanoma survivors about breast cancer risk and prevention. We were also among the first to evaluate the association between exposure to a specific class of chemotherapy agents and SMN risk in AYAs, which could not be done in registry-based studies.

## Conclusions

This study provided a detailed overview of SMN focusing on survivors of AYA cancers using an internal, individually matched comparison cohort without a history of cancer and characterized in detail the cumulative incidence function and risk factors for SMN among survivors of AYA cancer. Additional studies are needed to characterize SMN risk factors within specific pairs of first and second malignant neoplasm type to inform the development of tailored screening and prevention guidelines. Given the challenge of long-term follow-up of survivors of AYA cancer in a traditional cohort setting,^39^ retrospective analysis of members from integrated health systems with access to information on detailed treatment history, lifestyle, and genetic risk factors via electronic medical records may be one of the most promising approaches to further understand the role of treatment and other cancer risk factors in SMN development, especially when enhanced with linkage with large-scale registries or surveys.

## Notes

Supplement. **eTable 1.** Summary of Algorithms for Determining Multiple Primaries for Breast Cancer, Lymphoma, Melanoma and Testicular Cancer Based on the SEER Multiple Primary Rules **eTable 2.** Distribution of Types of Second Malignant Neoplasm Among Cancer Survivors and First Primary Cancer Among the Comparison Cohort Diagnosed During the Study Follow-up Period **eTable 3.** Risk Factors for SMN Among Survivors of AYA Cancer of the Five Most Common First Cancer Type—Multivariable Poisson Regression **eTable 4.** Hazard Ratio of Mortality Risk After Developing SMN in Cancer Survivors Compared With Mortality After Developing First Cancer in the Comparison Cohort **eFigure 1.** Study Population Flowchart **eFigure 2.** Cumulative Incidence Function of SMN Considering Competing Risk by First Cancer Type
