# Supplementary material for: Incidence, Risk Factors, and Mortality Associated With Second Malignant Neoplasms Among Survivors of Adolescent and Young Adult Cancer
Source: JAMA Netw Open. 2019 Jun 7;2(6):e195536. doi: 10.1001/jamanetworkopen.2019.5536 (PMC6563559; doi:10.1001/jamanetworkopen.2019.5536)
Supplement: Supplement. — eTable 1. Summary of Algorithms for Determining Multiple Primaries for Breast Cancer, Lymphoma, Melanoma and Testicular Cancer Based on the SEER Multiple Primary Rules eTable 2. Distribution of Types of Second Malignant Neoplasm Among Cancer Survivors and First Primary Cancer Among the Comparison Cohort Diagnosed During the Study Follow-up Period eTable 3. Risk Factors for SMN Among Survivors of AYA Cancer of the Five Most Common First Cancer Type—Multivariable Poisson Regression eTable 4. Hazard Ratio of Mortality Risk After Developing SMN in Cancer Survivors Compared With Mortality After Developing First Cancer in the Comparison Cohort eFigure 1. Study Population Flowchart eFigure 2. Cumulative Incidence Function of SMN Considering Competing Risk by First Cancer Type [file jamanetwopen-2-e195536-s001.pdf]

Supplementary Online Content

Chao C, Bhatia S, Xu L, et al. Incidence, risk factors, and mortality associated with second malignant neoplasms among survivors of adolescent and young adult cancer. *JAMA Netw Open*. 2019;2(6):e195536. doi:10.1001/jamanetworkopen.2019.5536

eTable 1. Summary of Algorithms for Determining Multiple Primaries for Breast Cancer, Lymphoma, Melanoma and Testicular Cancer Based on the SEER Multiple Primary Rules

eTable 2. Distribution of Types of Second Malignant Neoplasm Among Cancer Survivors and First Primary Cancer Among the Comparison Cohort Diagnosed During the Study Follow-up Period

eTable 3. Risk Factors for SMN Among Survivors of AYA Cancer of the Five Most Common First Cancer Type—Multivariable Poisson Regression

eTable 4. Hazard Ratio of Mortality Risk After Developing SMN in Cancer Survivors Compared With Mortality After Developing First Cancer in the Comparison Cohort

eFigure 1. Study Population Flowchart

eFigure 2. Cumulative Incidence Function of SMN Considering Competing Risk by First Cancer Type

This supplementary material has been provided by the authors to give readers additional information about their work.

© 2019 Chao C et al. *JAMA Network Open.*

eTable 1. Summary of algorithms for determining multiple primaries for breast cancer, lymphoma, melanoma and testicular cancer based on the SEER multiple primary rules

Cancer type	SEER multiple primary rules
Breast cancer	• ICD-O-3 topography codes different at the second (Cxxx) and/or third (Cxxx) character<br>• Diagnosed more than five (5) years apart<br>• Both breasts<br>• ICD-O-3 histology codes different at the first (xxxx), second (xxxx), or third (xxxx) number<br>https://seer.cancer.gov/tools/mphrules/2007_mphrules_manual_08242012.pdf (for all solid tumors)
Lymphoma	• Determination based on histology<br>https://seer.cancer.gov/archive/icd-o-3/hematopoietic_primaries.d03152001.pdf
Melanoma	• ICD-O-3 topography codes are different at the second (Cxxx), third (Cxxx) or fourth (Cxxx) character<br>• Different laterality<br>• ICD-O-3 histology codes are different at the first (xxxx), second (xxxx), or third (xxxx) number<br>• Diagnosed more than 60 days apart
Testicular cancer	• Both sides of a paired site<br>• Diagnosed more than one (1) year apart<br>• ICD-O-3 topography codes different at the second (Cxxx) and/or third (Cxxx) character<br>• ICD-O-3 histology codes are different at the first (xxxx), second (xxxx), or third (xxxx) number

© 2019 Chao C et al. *JAMA Network Open.*

eTable 2. Distribution of types of second malignant neoplasm among cancer survivors and first primary cancer among the comparison cohort diagnosed during the study follow-up period.

	Survivors of AYA cancer Total N=622	Comparison cohort Total N=3437
Central nervous system	15 (2.41%)	136 (3.96%)
Gastrointestinal system	70 (11.25%)	386 (11.23%)
Hodgkin lymphoma	4 (0.64%)	32 (0.93%)
Non-Hodgkin lymphoma	24 (3.86%)	107 (3.11%)
Bladder	5 (0.80%)	28 (0.81%)
Bone	7 (1.13%)	9 (0.26%)
Breast	198 (31.83%)	1116 (32.47%)
Cervix	6 (0.96%)	93 (2.71%)
Female genital	10 (1.61%)	39 (1.13%)
Leukemia	14 (2.25%)	60 (1.75%)
Lung	12 (1.93%)	85 (2.47%)
Male genital	1 (0.16%)	4 (0.12%)
Melanoma	85 (13.67%)	301 (8.76%)
Oropharynx	18 (2.89%)	68 (1.98%)
Other	25 (4.02%)	211 (6.14%)
Ovary	28 (4.50%)	96 (2.79%)
Prostate	16 (2.57%)	120 (3.49%)
Renal	19 (3.05%)	101 (2.94%)
Soft tissue	10 (1.61%)	27 (0.79%)
Testes	17 (2.73%)	36 (1.05%)
Thyroid	23 (3.70%)	218 (6.34%)
Uterus	15 (2.41%)	164 (4.77%)
**By solid tumor status**		
Solid	578 (92.93%)	3216 (93.57%)
Non-solid	44 (7.07%)	221 (6.43%)

AYA: adolescent and young adult

© 2019 Chao C et al. *JAMA Network Open.*

eTable 3. Risk factors for SMN among survivors of AYA cancer of the five most common first cancer type—Multivariable Poisson regression

	IRR (95% CI)	p-value
**Breast cancer survivors**		
Age at diagnosis, years		
15-29	Ref	
30-39	1.03 (0.59-1.78)	0.92
Race/ethnicity		
Non-Hispanic white	Ref	
Asian/Pacific Islanders	0.50 (0.27-0.93)	0.03
Non-Hispanic black	0.79 (0.51-1.22)	0.29
Hispanic	1.10 (0.78-1.54)	0.59
TNM Stage at diagnosis		
Stage I	Ref	
Stage II	0.94 (0.68-1.29)	0.69
Stage III/IV	1.06 (0.61-1.84)	0.83
Calendar year at diagnosis		
1992-2002	Ref	
2003-2014	0.61 (0.43-0.87)	0.01
Radiation therapy	1.52 (1.12-2.05)	0.01
**Melanoma survivors**		
Age at diagnosis, years		
15-29	Ref	
30-39	1.46 (0.90-2.37)	0.13
Female sex	1.11 (0.73-1.68)	0.62
Race/ethnicity		
Non-Hispanic white	Ref	
Other race/ethnicity	1.07 (0.55-2.08)	0.84
TNM Stage at diagnosis		
Stage I	Ref	
Stage II	1.37 (0.79-2.35)	0.26
Stage III/IV	2.83 (1.40-5.71)	0.004
Calendar year at diagnosis		
1992-2002	Ref	
2003-2014	0.89 (0.55-1.41)	0.61
**Lymphoma survivors**		
Age at diagnosis, years		
15-19	Ref	
20-29	0.58 (0.26-1.30)	0.18
30-39	0.87 (0.42-1.79)	0.70

© 2019 Chao C et al. *JAMA Network Open.*

Female sex	1.53 (0.86-2.71)	0.14
Race/ethnicity		
Non-Hispanic white	Ref	
Asian/Pacific islander	0.96 (0.29-3.20)	0.94
Non-Hispanic black	1.98 (0.94-4.17)	0.07
Hispanic	0.97 (0.49-1.90)	0.92
TNM Stage at diagnosis		
Stage I	Ref	
Not applicable	2.78 (0.35-22.12)	0.33
Stage II	1.52 (0.70-3.34)	0.29
Stage III/IV	1.94 (0.85-4.44)	0.12
Calendar year at diagnosis		
1992-2002	Ref	
2003-2014	0.50 (0.26-0.95)	0.03
Radiation therapy	1.85 (1.02-3.36)	0.04
**Testicular cancer survivors**		
Age at diagnosis, years		
15-29	Ref	
30-39	1.82 (0.80-4.10)	0.15
Non-Hispanic white race/ethnicity	0.96 (0.45-2.02)	0.91
TNM Stage at diagnosis		
Stage I	Ref	
Stage II	0.45 (0.10-1.94)	0.28
Stage III/IV	1.09 (0.39-3.01)	0.87
Calendar year at diagnosis		
1992-2002	Ref	
2003-2014	0.86 (0.39-1.90)	0.70
Radiation therapy	0.92 (0.42-2.05)	0.84
**Thyroid cancer survivors**		
Age at diagnosis, years		
15-19	Ref	
20-29	1.87 (0.23-14.96)	0.56
30-39	3.73 (0.51-27.43)	0.20
Female sex	1.47 (0.57-3.81)	0.42
Race/ethnicity		
Non-Hispanic white	Ref	
Asian/Pacific Islanders	1.54 (0.59-3.99)	0.37
Non-Hispanic black	0.59 (0.08-4.61)	0.62
Hispanic	1.77 (0.85-3.72)	0.13
TNM Stage at diagnosis		
Stage I	Ref	
Stage II	0.84 (0.25-2.78)	0.78

© 2019 Chao C et al. *JAMA Network Open.*

Stage III/IV	1.06 (0.32-3.48)	0.92
Calendar year at diagnosis		
1992-2002	Ref	
2003-2014	1.01 (0.53-1.93)	0.98

SMN: second malignant neoplasm; AYA: adolescent and young adult; IRR: incidence rate ratio; CI: confidence interval: Ref: reference category
Categories of certain covariates may vary between first cancer types due to small sample size in some subgroups.

© 2019 Chao C et al. *JAMA Network Open.*

eTable 4. Hazard ratio of mortality risk after developing SMN in cancer survivors compared with mortality after developing first cancer in the comparison cohort.

Survivors of AYA cancer			Comparison cohort			
# death	Number with SMN	Incidence per 1,000 p-yr	# deaths	Number with first cancer	Hazard ratio[a] (95% CI)	P-Value
188	622	52.80	581	3437	1.90	1.61

AYA: adolescent and young adult; SMN: second malignant neoplasm; p-yr: person-years; CI: confidence interval
[a]Adjusted for age, gender and race/ethnicity.

© 2019 Chao C et al. *JAMA Network Open.*

eFigure 1. Study population flowchart

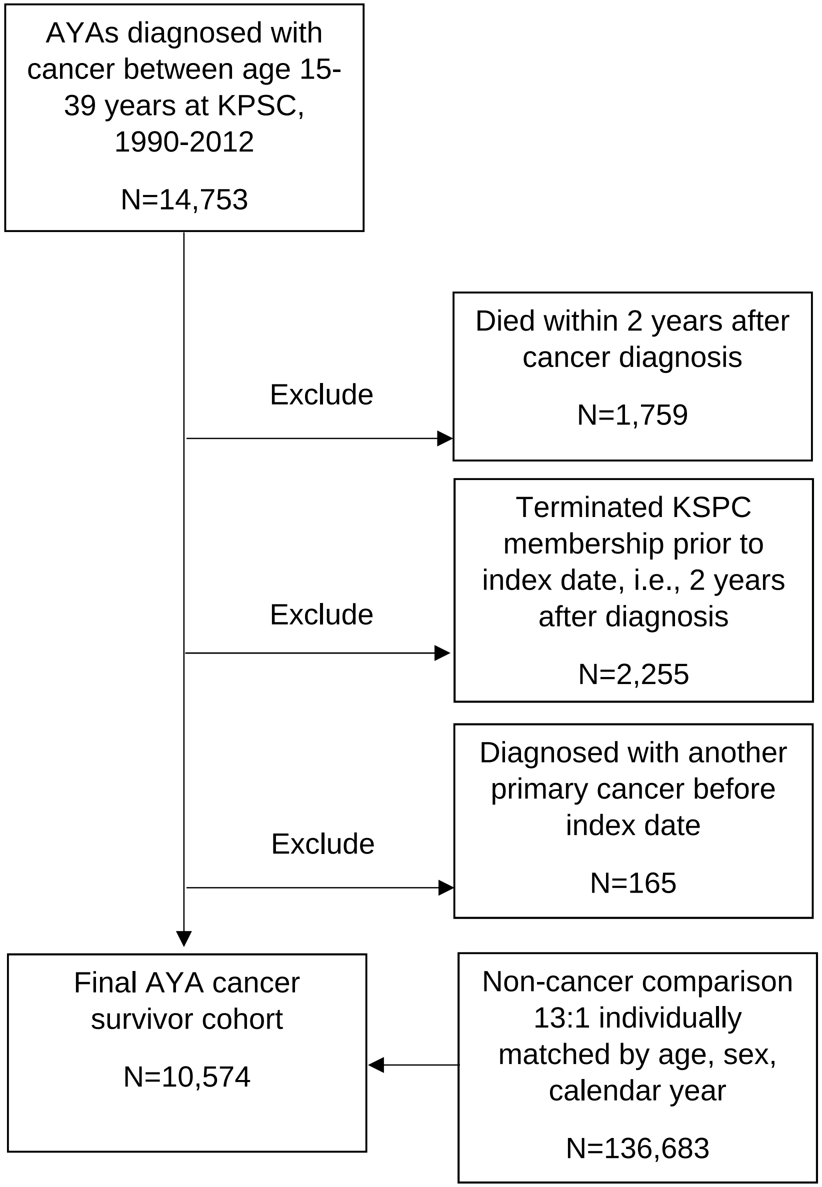

© 2019 Chao C et al. *JAMA Network Open.*

eFigure 2. Cumulative incidence function of SMN considering competing risk by first cancer type

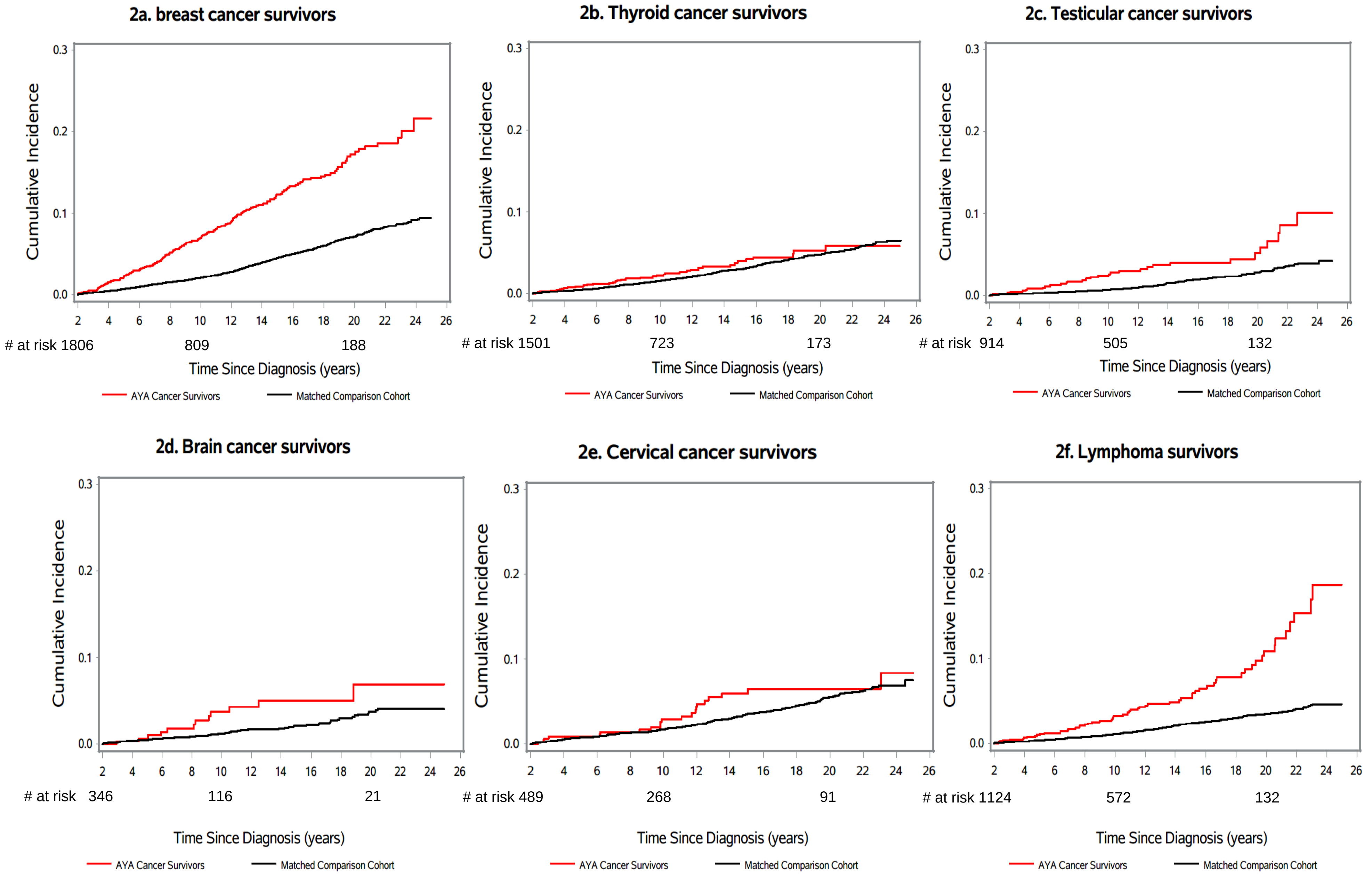

© 2019 Chao C et al. *JAMA Network Open.*

2g. Melanoma survivors

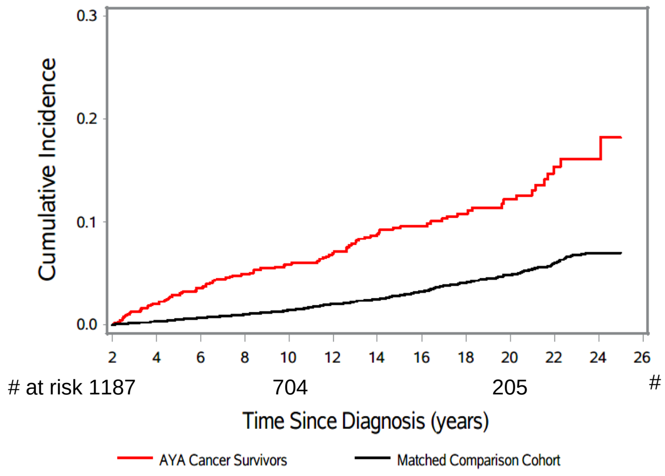

2h. Ovarian cancer survivors

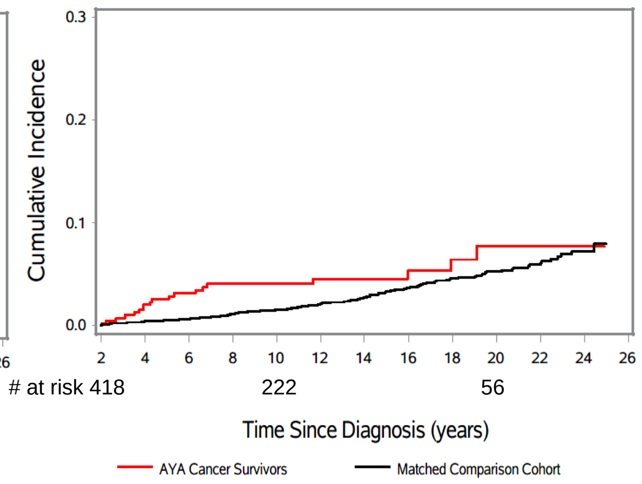

© 2019 Chao C et al. *JAMA Network Open.*
